# Asymmetric practices of reading and writing shape visuospatial attention and discrimination

**DOI:** 10.1038/s41598-020-78080-0

**Published:** 2020-12-03

**Authors:** Rita Mendonça, Margarida V. Garrido, Gün R. Semin

**Affiliations:** 1 William James Center for Research, ISPA – Instituto Universitário, Rua Jardim Do Tabaco, 34, 1149-041 Lisbon, Portugal; 2 Iscte, Instituto Universitário de Lisboa, Cis-Iscte, Av. Das Forças Armadas, 1649-026 Lisbon, Portugal; 3 Faculty of Social and Behavioral Sciences, Utrecht University, 3584 CS Utrecht, The Netherlands

**Keywords:** Psychology, Human behaviour

## Abstract

Movement is generally conceived of as unfolding laterally in the writing direction that one is socialized into. In ‘Western’ languages, this is a left-to-right bias contributing to an imbalance in how attention is distributed across space. We propose that the rightward attentional bias exercises an additional unidirectional influence on discrimination performance thus shaping the congruency effect typically observed in Posner-inspired cueing tasks. In two studies, we test whether faces averted laterally serve as attention orienting cues and generate differences in both target discrimination latencies and gaze movements across left and right hemifields. Results systematically show that right-facing faces (i.e. aligned with the script direction) give rise to an advantage for cue-target pairs pertaining to the right (versus left) side of space. We report an asymmetry between congruent conditions in the form of right-sided facilitation for: (a) response time in discrimination decisions (experiment 1–2) and (b) eye-gaze movements, namely earlier onset to first fixation in the respective region of interest (experiment 2). Left and front facing cues generated virtually equal exploration patterns, confirming that the latter did not prime any directionality. These findings demonstrate that visuospatial attention and consequent discrimination are highly dependent on the asymmetric practices of reading and writing.

## Introduction

Visual scanning of space is not a random process but reveals different systematic biases^1,2^. One such bias is driven by cultural habits that are associated with reading and writing practices^3^. This bias in movement of visual scanning unfolds laterally in the direction of the writing and reading practices of the language one is socialized in. In the case of Western cultures, this is a left-to-right bias. Such culturally established habits modulate the spatial order and consequently underlie the representation of space^4,5^. The objective of the current studies is to examine whether the rightward attentional bias driven by the culturally anchored direction of movement influences the congruency effect typically observed in Posner-inspired cueing tasks. The congruency effect shows that when cue indication and target location are consistent, performance (e.g., target discrimination latency) is enhanced. It is hindered when cue indication and target location are inconsistent^6^. The central and novel feature of the two experiments reported here, is twofold: the conjunction of head orientation and gaze as directional spatial cues, namely faces averted laterally (to the right or the left), and a highly demanding discrimination task which prompts automatic responses within a brief response interval. We expected an asymmetry for congruent face cue-target combinations. The prediction was that faces laterally averted rightward as cues (i.e. congruent with script direction) would be consistent with the overlearned left-to-right trajectory and therefore advantage target discrimination and eye-gaze movements to targets located at the right compared to the condition with leftward averted faces as cues.

In the following, we present first the background to the current research, namely the ‘spatial agency bias’ (SAB, for a review see^3^) along with the research it has generated on attentional and cognitive processing. Subsequently, we briefly present the rationale of the methodological approach we used for the spatial cueing tasks. Finally, an overview of the two experiments is provided.

### Spatial agency bias

There is to date considerable evidence that the mental models we use to navigate the social world, anticipate actions, make predictions, reason and solve problems are biased by the cultural convention of text direction^7^. This assumption is largely anchored in the spatial agency bias model. Because in ‘Western’ languages script direction, and therefore motion, unfolds rightward people favor representations in which the agent is spatially positioned to the left of the recipient of the action^8^. There is also a preferential directionality when representing social groups in space, with stereotypically agentic groups (e.g., males, young people) being systematically placed to the left of groups with less agentic qualities (e.g., females, old people)^9^. The opposite holds for languages such as Arabic and Hebrew where the use of spatial information is reversed, that is, action progresses from right to left. However, this effect is considerably weaker in cultures where writing is leftward^10^, likely due to the existing exposure to westernized spatial layouts whereas exposure to leftward cultures in the West is virtually nonexistent.

These habitualized asymmetric practices permeate a wide range of attentional and cognitive processes well beyond the activities of reading and writing. For instance, script direction affects the mental representation of time^11–13^, political landscape^14,15^, numerical magnitude (SNARC effect^16^), or ordinal and action sequences both in adults^17,18^ and preverbal infants^19^. Importantly, these overlapping regularities derived from the left-to-right movement continuum do not stem from language and symbolic knowledge acquisition alone but instead are assimilated throughout continuous exposure to everyday activities (e.g., bookshelf organization^20^; visual representation on Websites^21^).

A commonly reported phenomenon in attention-demanding visual tasks is the unequal distribution of visuospatial attention towards the left side of space (i.e. pseudoneglect^22^) resulting, for instance, in the misbisection of a horizontal line with significant leftward deviation of veridical midpoint^23^. While the left hemispace bias is likely the product of right hemispheric specialization for visuospatial attention^24^, virtually all studies investigating left biases in spatial attention report a subgroup of rightward biased individuals^22^. What is interesting is that the preference for the left hemispace can be modulated in the opposite direction when tasks are performed by readers from right-to-left speaking countries^25,26^. This indicates that cultural factors such as native reading direction may, at the very least, attenuate to a certain extent the predisposition to overattend to the left side of space. Thus, culturally and biologically-determined accounts of the laterality of the visual attention system are not mutually exclusive but complementary instead, as this imbalance in visual attention is likely a combination of a person’s primary writing system and hemispheric specialization^19,27,28^.

Indeed, a solid corpus of research has shown that reading and writing scanning habits produce a critical left-anchoring tendency in scanning strategies^29–31^. In an early report, using gaze-contingent moving windows, Pollatsek and colleagues^32^ found that participants deployed visual attention to the right while a mirror reversal was found for participants reading in Hebraic. Furthermore, task performance is enhanced when target stimuli flow in a script-coherent direction because one is able to anticipate the occurrence of future information and predict where a moving target will end up^33,34^. Likewise, in a serial visual search task, left-to-right readers exhibited more accurate detections for the right hemifield and right-to-left readers for the left hemifield^35^. Notably, there was negligible lateralization for bidirectional readers of English and Farsi. In the same vein, in a series of eye-tracking experiments, Afsari and colleagues^25^ reported bilinguals from languages with opposing scripts to display flexibility in changing the direction of the spatial bias according to the type of text they read prior to freely exploring images. Hence, if attentional biases can be mitigated through exposure to distinct scanning habits, a culturally based account must be at the core of preferences in spatial exploration.

### Overview of the methodology

Visuospatial attention has been examined with variations of Posner-inspired cueing tasks^6,36,37^. It is well-established that when cue indication and target location are consistent, performance is enhanced, and hindered when cue indication and target location are inconsistent^38^. While this pattern holds for most studies, cue stimuli combining both head orientation and gaze direction can be powerful in Posner-inspired cueing tasks because it is well known that humans are positively tuned to lock onto others’ gaze. Indeed, both gaze direction and face orientation have long been used as directional prime cues to investigate visual performance (for a review see^39^). Human faces have been shown to be remarkably reliable triggers of attention shifts^40,41^. For example, Driver and colleagues^36^ found centrally presented face and gaze cues to evoke faster discrimination of peripheral target letters on the side the face gazed towards, even though participants were told the targets were four times more likely to appear on the opposite side. However, studies relying on faces cues have inspected gaze as a precursor of social interaction, focusing on the effects of, for instance, direct versus averted gaze^42^, or the combined effect of gaze direction and facial expression on cueing spatial attention^43^. The joint influence of combined gaze and head orientation and their implied directional flow on target discrimination is still scarce.

Because faces are distinctive due to their biological relevance, they trigger attentional shifts and facilitate processing at congruent locations even without awareness^44,45^. Eyes and attention continuously shift together in space and are indeed biased by the direction signaled by gaze^46^. For instance, Mansfield and colleagues^47^ reported spontaneous saccades following an averted gaze cue but prior to target onset which reinforces their automatic nature. Similarly, face cues but not arrows produce less accurate saccades to a target in incongruent trials, relative to congruent ones^48^. This vouches for the unique role of the face and eyes in automatically activating a similar motoric program in the participant by mere observation^49–51^.

Faces and their gaze direction acquire added significance in the context of Posner-inspired cueing tasks. Recent research^51^ has shown that rightward oriented faces imply agency from left to right in line with the culturally established writing-reading system. This would suggest that face gazes as cues in a Posner-inspired cuing task would have an asymmetrical attentional influence. Rightward oriented faces would be expected to facilitate a stronger rightward attentional shift compared to a leftward attentional shift with oriented faces as cues.

### Overview of the present experiments

Building upon the above line of research, we expected the reading and writing practices derived from the culturally established script direction to drive a biased scanning of external space. Across two studies we investigated the extent to which automatic attention orienting and subsequent target discrimination were shaped by visual social cues (i.e. faces presenting three distinct perspectives: left-facing, front-facing, right-facing). If the spatial bias found in the West is an attention-driving mechanism, then the robust congruency effect typically found in cueing tasks should be amplified for right-cue/right target pairs, over left-cue/left target pairs. This bias would be expected as a result of a culturally habitualized script effect and conflict with the prediction that symmetrical outcomes would result from a congruent target prime constellation, namely left-left and right-right.

In experiment 1, we relied on behavioral measures, namely response times, to investigate if distinct face orientations serve as attention orienting cues and affect target discrimination latencies. In experiment 2, we extended previous findings and introduced an objective process measure of eye movement to address the underlying processes that drive discrimination decisions.

## Experiment 1

In experiment 1, photos of faces were presented when they were facing left vs. front vs. right in the middle of the monitor screen. These photos were chosen to serve as primes driving attention orienting cues and were expected to shape discrimination decisions in line with the SAB. We expected: 1) Shorter response times when the face position was congruent with the target letter position as compared to when face and target letter positions were incongruent; 2) The effect on 1) was expected to be amplified when cue-target pairs referred to the right, that is, right-facing (left-facing) primes were expected to produce shorter reaction times, when the target letter appears in the right (left) visual field; 3) No difference in discrimination latencies for targets on the left and right visual fields were expected when the prime was front-facing, and thus constituted the baseline condition; 4) In the absence of a target letter within the target sets, false detections should be congruent with the face position.

### Results

#### Reaction time and false detections

Data for correct response times were analyzed. For obvious reasons, the no-target trials were not included in this analysis. In order to control for anticipatory and spurious responses, reaction times under 100 ms were excluded. A residual percentage (8.57%) of missing responses was observed across all data points.

We performed a linear mixed model analysis (LMM) which allowed us to control for the variance in response time introduced by the photo stimuli and the participants’ individual differences (photo ID and participant ID). No severe violation of the homoscedasticity and normality principles were observed in the residual plots.

The LMM was conducted including the photo ID and participant ID as clustering factors, the reaction time as the dependent variable, and face orientation (left vs. front vs. right), target letter (left vs. right), and response interval (700 ms vs. 1000 ms vs. 1300 ms) as categorical independent variables. As fixed effects in the model, we considered the face orientation, target letter, and response interval as well as their second and third-order interactions. As random effects, we included random intercepts per participant and per photo. Moreover, the model was estimated using restricted maximum likelihood, and a Satterthwaite approximation of the degrees of freedom was considered. The LMM analysis was performed using the GAMLj module^52^ implemented with the jamovi software^53^.

The LMM analysis (*R*^2^_*marginal*_ = 0.15; *R*^2^_*conditional*_ = 0.28) revealed a main effect of face orientation, *F* (2, 4255) = 37.24, *p* < 0.001, target letter, *F* (1, 4261) = 77.90, *p* < 0.001, and response interval, *F* (2, 4257) = 292.87, *p* < 0.001. Post-hoc comparisons with Bonferroni correction revealed that front-facing photos and right-facing photos (*M* = 542, *SE* = 12.7 and *M* = 545, *SE* = 12.6, respectively) gave rise to significantly shorter reaction times than left-facing primes (*M* = 596, *SE* = 12.7, *p*’s < 0.001). Different discrimination times were not observed between front-facing and right-facing faces (*p* = 1.000). Interestingly, the 700 ms interval (*M* = 476, *SE* = 12.8) produced faster response times than the 1000 ms and 1300 ms response intervals (*M* = 562, *SE* = 12.6 and *M* = 646, *SE* = 12.5, respectively; *p*’s < 0.001). Response times in the 1000 ms window were also significantly faster than those in the 1300 ms window (*p* < 0.001). Thus, the shorter the response window, the faster the discrimination. Additionally, target letters embedded on the right visual field (*M* = 536, *SE* = 12.4) were detected significantly faster than those on the left side (*M* = 586, *SE* = 12.3, p < 0.001).

**Figure 1:**
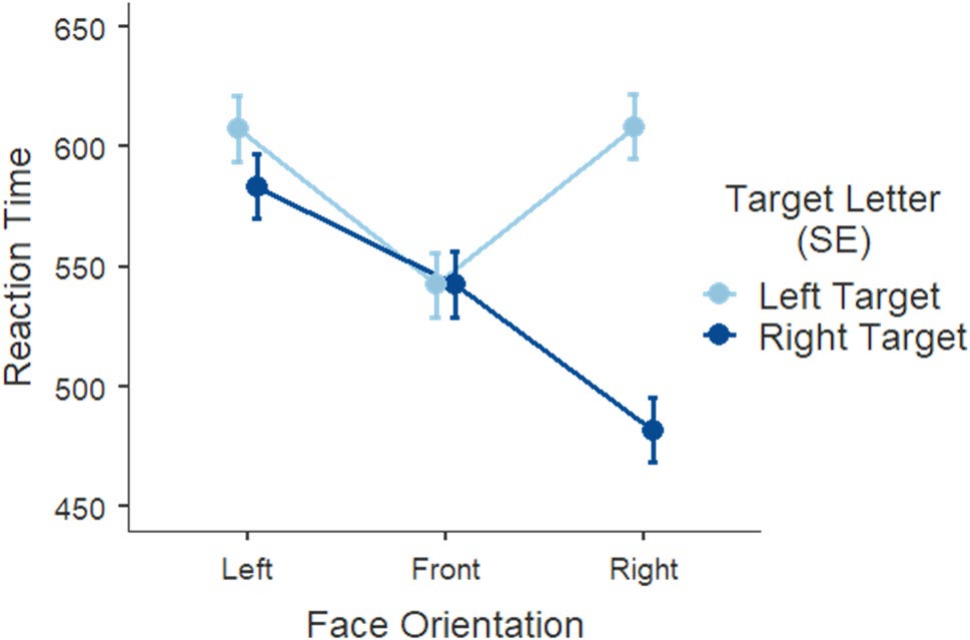
Mean response time (in milliseconds) as a function of face orientation of the cue, and target letter position. Error bars represent the standard error from the mean.

We then turned to the second order interaction between face orientation and target location as it addresses directly our first prediction that congruent (vs. incongruent) cue-target pairs would improve discrimination performance, *F* (2, 4255) = 47.50, *p* < 0.001 (Fig. 1). As expected, right oriented faces gave rise to significantly faster discrimination of targets on the right side (*M* = 482, *SE* = 13.5) relative to those on the left side of space (*M* = 608, *SE* = 13.6, *p* < 0.001). Front-facing faces did not generate different discrimination latencies across left (*M* = 542, *SE* = 13.5) and right presented targets (*M* = 543, *SE* = 13.6, *p* = 1.000), nor did left-facing faces (left target: *M* = 608, *SE* = 13.5; right target: *M* = 584, *SE* = 13.7, *p* = 0.216). We thus failed to observe the predicted effect on congruency pertaining leftward faces as the generated discrimination latencies did not statistically differ across target locations.

Additionally, and to address our second prediction of an imbalance in performance favoring the right(left)-sided targets following rightward (leftward) faces, we report the post-hoc comparison in discrimination latencies between left face – left target vs. right face – right target conditions. Results show a clear and significant asymmetry in performance between the two congruent conditions and favoring rightward cue-target pairs, *t* (4257) = 12.9945, *p* < 0.001.

A three-way interaction between face orientation, target letter and interval was also obtained, *F* (4, 4255) = 3.94, *p* = 0.003, although no predictions were made regarding the influence of the three time intervals on discrimination.

To ensure that hand dominance did not account for the observed rightward facilitation, we performed a paired samples t-test comparing the average reaction time of the *q* key press (*M* = 552.34, performed with the left index finger) and the *p* key press (*M* = 563.91, performed with the right index finger) across participants. Response latencies were not different when selecting *q* and *p* keys, *t* (44) = -1.660, *p* = 0.104, confirming that handedness did not drive the benefits on the right hemifield.

To investigate the trials without a target letter within the sets, we analyzed what we have called false detections (i.e. the selection of the left or right response keys) as predicted by the head orientations and the response intervals. To this end, given the dichotomous nature of the dependent variable, we performed a logistic mixed effects model (generalized mixed linear model for binomial outcomes). The present model predicted the proportion of the selection of the response key (0 = left key; 1 = right key) in terms of log odds with head orientation (left face vs. front face vs. right face) and response interval (700 ms vs. 1000 ms vs 1300 ms), and head orientation x response interval interaction as fixed effects. As cluster variables and random effects, we included random intercepts for participant ID and photo ID to control for dependencies in key selection driven by the variance introduced by the photos as well as the participants’ interindividual differences. The logistic mixed effects model was performed using the GAMLj module^52^ implemented with the jamovi software^53^. The model (n = 45; *R*^2^_*marginal*_ = 0.11; *R*^2^_*conditional*_ = 0.14) revealed that the probability of selecting a response key is different between head orientations (χ^2^ = 153.308, df = 2, *p* < 0.001). In contrast, the selection of the response key was not influenced by response interval (χ^2^ = 0.634, df = 2, *p* = 0.728) nor by the interaction head orientation x response interval (χ^2^ = 2.688, df = 4, *p* = 0.611).

We investigated the fixed parameters estimates for head orientation assuming left faces as the reference category. Positive regression slopes for both face comparisons indicate that, relative to left faces, front faces (*β* = 0.25, *SE* = 0.11, *z* = 2.216, *p* = 0.027) and right faces (*β* = 1.47, *SE* = 0.12, *z* = 11.853, *p* < 0.001) have a greater likelihood of inducing false detections to the right, or inducing right key selection. By attending to the odds ratio, we can have a better perception of what these coefficients represent. The odds of a participant scoring 1 on the selection of response key, that is of pressing the right key, increases by a factor of 1.28 (CI [1.029, 1.59]) in frontal faces (compared to left faces). The influence of right faces can be seen in the odds ratio indicating that participants are 4.33 times more likely to press the right key (CI [3.398, 5.52]) in right faces relative to left faces. The remaining coefficients were not statistically significant (see Table 1 for detail on fixed effects parameter estimates).

**Table 1:** Fixed effects parameter estimates for the logistic mixed model predicting the proportion of false detections by head orientation and response interval.

	Effect	*B*	*exp(B)*	95% Confidence interval		*Z*
				Lower	Upper	
Head Orientation1	Front–Left^a^	0.2460*	1.279	1.029	1.59	2.216
Head Orientation2	Right–Left	1.4656***	4.330	3.398	5.52	11.853
Response Interval1	1000–700^b^	0.0446	1.046	0.848	1.29	0.418
Response Interval2	1300–700	− 0.0387	0.962	0.778	1.19	− 0.357
Head Orientation1 × Response Interval1	Front–Left × 1000–700	0.1942	1.214	0.756	1.95	0.804
Head Orientation2 × Response Interval1	Right–Left × 1000–700	− 0.1587	0.853	0.501	1.45	− 0.584
Head Orientation1 × Response Interval2	Front–Left × 1300–700	0.2928	1.340	0.831	2.16	1.200
Head Orientation2 × Response Interval2	Right–Left × 1300–700	0.0680	1.070	0.626	1.83	0.249

^a^Left is the reference category for the head orientation variable; ^b^700 ms interval is the reference category for the response interval variable.

*p < .05 ***p < .001.

**Figure 2:**
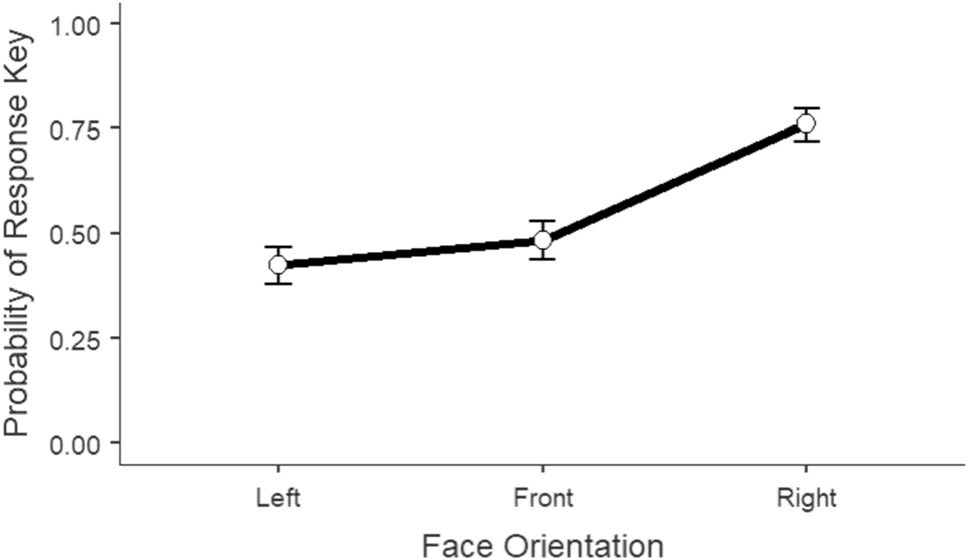
Proportion of false detections (0 = left key, 1 = right key) predicted by head orientation. Error bars represent 95% CI’s.

Finally, we explored post hoc comparisons for head orientation using the Bonferroni correction procedure (Fig. 2). The probability of selecting the right key in front faces (0.48) is significantly lower than in right faces (0.76; *z* = -9.92, *p* < 0.001). The probability of selecting a right key in left faces (0.42) does not differ from selecting the right key in front faces (*z* = -2.22, *p* = 0.080), suggesting that left and frontal head perspectives induce similar probability patterns of false detections. Critically, the probability of selecting the right key following the presentation of left faces is significantly more reduced than following right faces (*z* = -11.85, *p* < 0.001).

Thus, and predicted by our fourth hypothesis, in the absence of a target letter, false detections seem to be congruent with the face position. Specifically, the proportion of false detections on the left and right keys is significantly predicted by head orientation in that right faces induce right key clicking with a greater probability. Left and front faces are thus more likely to induce left key clicking, which is congruent with the direction they point towards (baseline faces lack directionality and are therefore expected to resemble performance for left faces). False detections seem to complement what was observed in response times by confirming that face primes do influence attention allocation towards their implied directionality. Taken together with the right-sided advantage observed in response latencies, false detections speak to the habitualized left-to-right eye trajectory and the underlying rightward bias it generates.

## Experiment 2

This experiment extended the previous results and provided process evidence by tracking the participants’ eye movements while performing the face cueing task. We measured overt attention (i.e. attention accompanied by oculomotor movements). Cue, target stimuli, and procedure were identical to Experiment 1. The design had two minor modifications: only the 1000 ms response interval was kept, and the target-absent condition was removed. This resulted in a 3 (Face orientation: left vs. front vs. right) × 2 (Target letter position: left vs. right) within participants manipulation. All measures, manipulations, and exclusions in this study are reported.

Aside from the expected replication of the previous experiment (i.e. response time), our main predictions were: 1) Head position would drive initial gaze movement, that is, the direction of the first saccade should be consistent with the face orientation of the prime; 2) When the face position is to the front, the automatic gaze direction is expected to be to the left. This is based on the assumption that front faces do not prime any directionality, hence they should induce the habitualized attention scanning path, starting from the left and moving to the right; 3) Earlier onset time for the first fixation in AOI for congruent compared to incongruent conditions, with an advantage for cue-target pairs pertaining to the right.

### Results

#### Reaction time

Data for correct response times were analyzed. Discriminations shorter than 100 ms were removed from the analysis. Missing responses were 21.47% across all data. To control for the variance introduced by the models’ photos (photo ID), as well as the participants’ interindividual differences (participant ID), a LMM was conducted. A visual inspection of the residual plots did not reveal any severe violation of the homoscedasticity or normality assumptions. The LMM was conducted including the photo ID and participant ID as clustering factors, the reaction time as the dependent variable, and face orientation (left vs. front vs. right), and target letter (left vs. right) as categorical independent variables. As fixed effects in the model, we considered the face orientation and target letter, as well as their interaction. As random effects, we included random intercepts per participant and per photo. The model was estimated using restricted maximum likelihood, and a Satterthwaite approximation of the degrees of freedom was considered.

**Figure 3:**
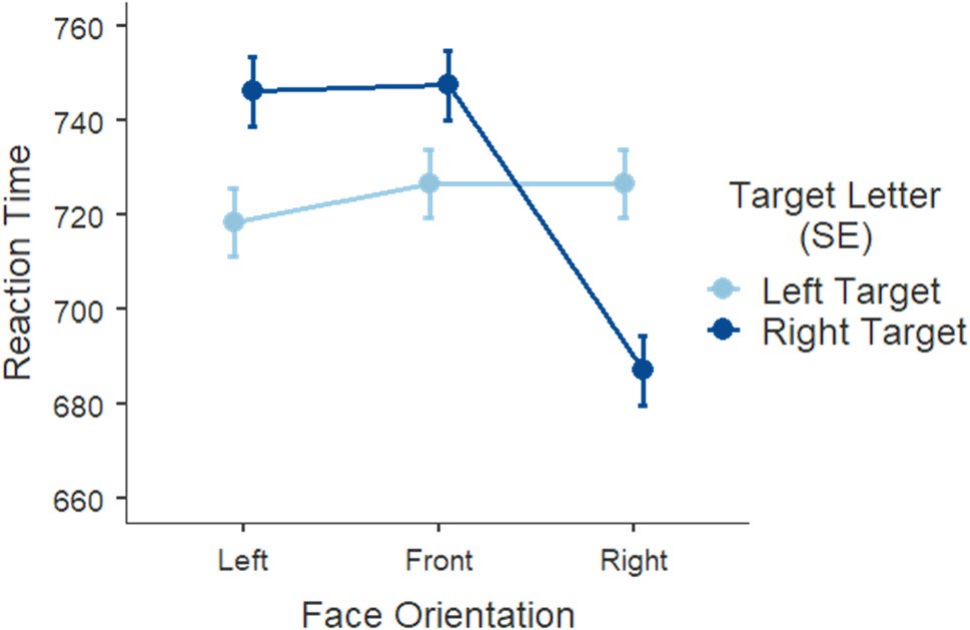
Mean response time (in milliseconds) as a function of face orientation of the cue and target letter position. Error bars represent the standard error from the mean.

The LMM (*R*^2^_*marginal*_ = 0.02; *R*^2^_*conditional*_ = 0.08) showed a main effect of face orientation, *F* (2, 5407) = 23.827, *p* < 0.001, with significantly faster responses following right-facing cues (*M* = 707, *SE* = 6.55) than left (*M* = 732; *SE* = 6.55; *p* < 0.001) and front-facing cues (*M* = 737, *SE* = 6.50; *p* < 0.001). Response time following front and left-facing cues was not significantly different (*p* = 0.918). The interaction face orientation x target location, *F* (2, 5409) = 30.585, *p* < 0.001, revealed faster discrimination when cue and target where spatially congruent (Fig. 3). Following a left-facing prime cue, target letters on the left (*M* = 718, *SE* = 7.29) were detected faster than targets on the right (*M* = 746, *SE* = 7.44; *p* < 0.001). Once again, following a right-facing face, this mean difference was amplified for the discrimination of targets on the right (*M* = 687, *SE* = 7.48) relative to the left (*M* = 727, *SE* = 7.25; *p* < 0.001). Frontal, neutral faces have resembled the pattern obtained for left-facing photos, albeit less pronounced. That is, front faces produced shorter response latencies on the left (*M* = 727, *SE* = 7.19) compared to the right side of space (*M* = 747, *SE* = 7.36; *p* = 0.023).

In order to investigate whether response times replicated the asymmetry in congruent conditions observed in Experiment 1, we report the post-hoc comparison for the performance between left face – left target and right face – right target conditions. Response times did replicate the results obtained in Experiment 1, *t* (5423) = 4.6152, *p* < 0.001, in that right targets following rightward faces were detected substantially faster than left targets following leftward faces.

Finally, we tested if handedness could have driven the obtained results. We compared averaged response times across participants when selecting the *q* key (*M* = 733.30, responded with the left finger) and the *p* key (*M* = 730.45, responded with the right finger), the latter coinciding with the dominant hand of most of the participants. We observed no differences in response times as a function of the hand used to respond, *t* (39) = -0.520, *p* = 0.606.

#### Direction of first saccade

To examine initial gaze movement, that is, whether the face primes induced the expected orientation of attention, we analyzed the proportion of the first saccade made in each trial to the left and right sides of space as a function of the orientation of the face. The first saccade, as well as all the remaining gaze measures, were recorded from the moment the head cue had elapsed. The onset point for the first saccade was controlled for the screen’s midpoint. The data were analyzed using a logistic mixed effects model. The model predicted the probability of the direction of the first saccade (0 = saccade to the left; 1 = saccade to the right) in terms of log odds. Head orientation (left face vs. front face vs. right face) was entered as a fixed effect. Participant ID and photo ID were included as random effects.

The model (n = 40; *R*^2^_*marginal*_ = 0.03; *R*^2^_*conditional*_ = 0.20) revealed that first saccades were not randomly distributed across left and right space, that is, they were shaped by the head orientation of the faces (χ^2^ = 238, df = 2, *p* < 0.001). Taking left faces as the reference category, we can observe that front faces are less likely to induce rightward saccades although not significantly so (*β* = -0.03, *SE* = 0.05, *z* = -0.569, *p* = 0.570). Conversely, the positive regression slope for right faces, compared to left faces, suggests that these face primes are more likely to trigger saccades to the right (*β* = 0.70, *SE* = 0.05, *z* = 13.106, *p* < 0.001). In fact, the odds ratio indicates that right (vs. left) faces increase the likelihood of initially looking towards the right by a factor of 2 (CI [1.807, 2.23]). Detailed parameter estimates can be found in Table 2.

**Table 2:** Fixed effects parameter estimates for the logistic mixed model predicting the proportion of the direction of the first saccade by head orientation.

	Effect	*B*	*exp(B)*	95% Confidence interval		*z*
				Lower	Upper	
Head Orientation	Front–Left	− .0299	.971	.875	1.08	− .569
Head Orientation	Right–Left	.6959***	2.006	1.807	2.23	13.106

Left is the reference category for the head orientation variable.

***p < .001.

**Figure 4:**
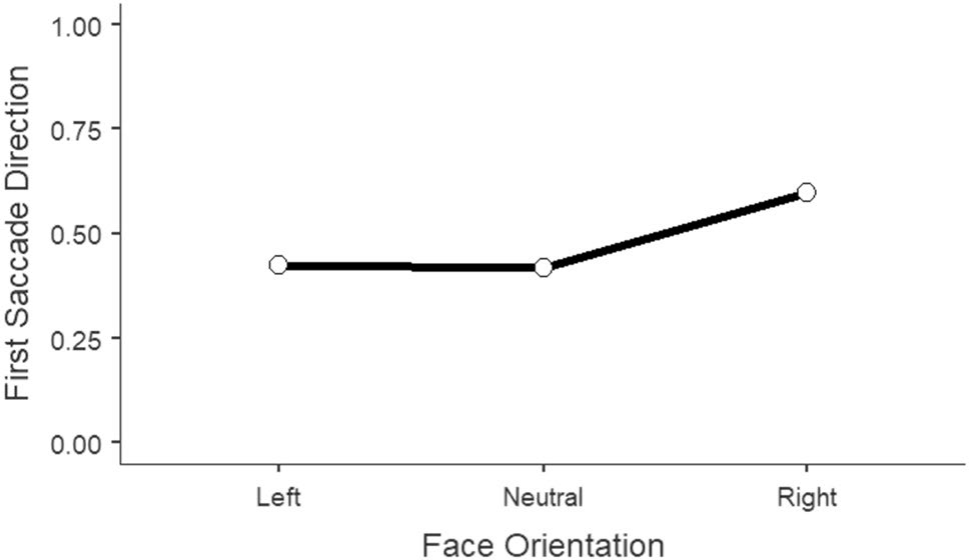
Proportion of the first saccade direction (0 = left saccade, 1 = right saccade) predicted by head orientation. Error bars represent 95% CI’s.

The post hoc comparisons using the Bonferroni procedure confirm that front faces have a lower probability (0.42) of giving rise to saccades to the right than right faces (0.60; *z* = -13.657, p < 0.001). Likewise, left faces (0.42) are also less likely to induce saccades to the right than right faces (*z* = -13.106, *p* < 0.001). Mimicking the pattern observed in the measure of reaction time, front-facing faces resembled the initial gaze distribution observed for left-facing faces (*z* = 0.569, *p* = 1) (Fig. 4).

#### First fixation onset

In the previous analysis we confirmed that face primes induce attention towards their implied directionality. The next analysis was to test if there is a rightward attentional bias driving gaze movement. To observe this habitualized bias we tested the time to first fixation which indicates how early in the trial participants’ gaze lands on the left and right located interest areas. Trials on which the eye-tracker lost track of the eye position were discarded (1.8% across conditions). We defined two rectangular areas of interest (AOI) corresponding to the two five-letter target sets (left AOI, right AOI) subtending the degrees of visual angle mentioned above.

We performed a LMM including the photo ID and the participant ID as clustering factors, the first fixation onset as the dependent variable, and face orientation (left vs. front vs. right), and AOI (left vs. right) as categorical independent variables. As fixed effects in the model, we considered the face orientation and AOI, as well as their interaction. As random effects, we included random intercepts per participant and per photo. The model was estimated using restricted maximum likelihood, and a Satterthwaite approximation of the degrees of freedom was considered.

**Figure 5:**
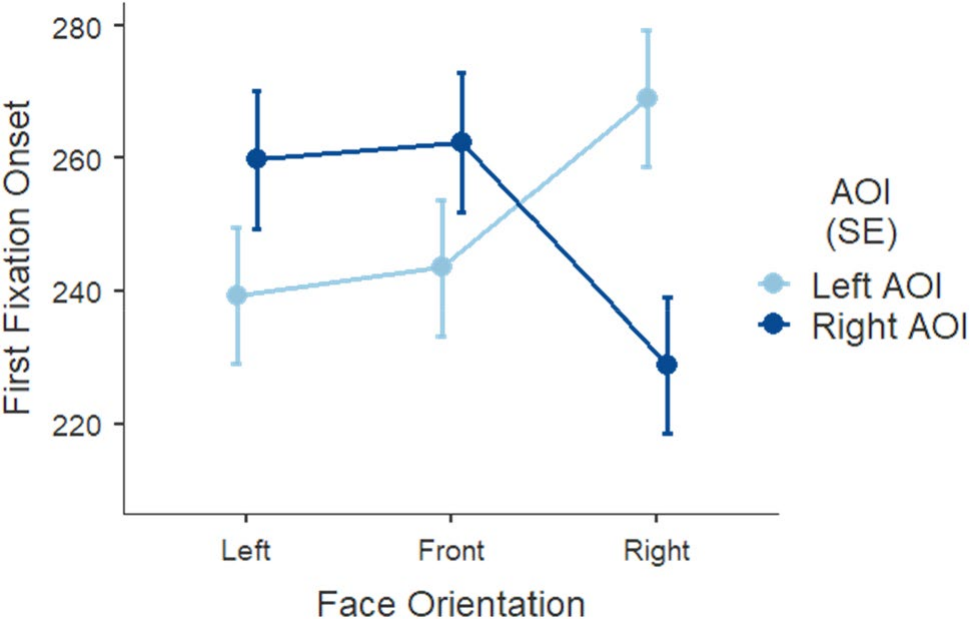
Mean time to first fixation (in milliseconds) as a function of face orientation of the cue and AOI. Error bars represent the standard error from the mean.

The LMM (*R*^2^_*marginal*_ = 0.001; *R*^2^_*conditional*_ = 0.20) revealed a significant interaction between face orientation and AOI, *F* (2, 8076) = 39.76042, *p* < 0.001 (Fig. 5). Both left and frontal face perspectives produced a similar pattern regarding the time it took to land the first fixation on the AOI around the target sets. This difference was more salient for left faces which generated earlier first fixations on the congruently located target area, that is the left AOI compared to the right AOI (*M* = 239, *SE* = 10.2; *M* = 260, *SE* = 10.4, respectively; *p* < 0.001). Front faces also gave rise to a slight advantage for earlier fixations on the left AOI (*M* = 244, *SE* = 10.2) than on the right AOI (*M* = 262, *SE* = 10.5; *p* = 0.004). As hypothesized, following a face cue averted rightward, participants attended earlier to the right AOI (*M* = 229, *SE* = 10.2) relative to the left AOI (*M* = 269, *SE* = 10.4; *p* < 0.001). In fact, participants attended the right AOI earliest, when the cue that preceded was a face oriented rightward, which is in line with our hypothesis.

The direct comparison of time to first fixation did not differ for fixations landing on the left AOI following left faces versus fixations landing on the right AOI following right face cues, *t* (8048) = 2.318, *p* = 0.307.

A complementary LMM analysis to the average velocity of the first saccade by face orientation (left vs. front vs. right) and saccade direction (left vs. right) revealed converging results, *F* (2, 10,021) = 10.596, *p* < 0.001.

## Discussion

In the present set of studies, we found visual performance to benefit from the habitualized reading and writing practices. As predicted, faces and their gaze direction as cues were found to have an asymmetrical attentional influence. Rightward oriented faces facilitated a stronger rightward attentional shift compared to the leftward attentional shift manifested with leftward oriented faces as cues. The two studies underscore the premise that rightward stimuli (i.e. aligned with script direction) preferentially engage human attention, hence triggering both biased discrimination latencies (experiments 1–2) and oculomotor movements (experiment 2).

In Experiment 1, which relied on behavioral measures alone, we did not observe the typical congruency effect on target detection speed reported in cueing tasks. Instead, irrespective of the time window for a response, only rightward faces gave rise to shorter latencies on right-located targets, relative to left-located ones. Contrary to an overwhelming body of literature showing otherwise (for a review see^54^), leftward faces did not generate the expected advantage in response times for targets located on the left hemifield. That is, they did not accelerate search time for left-sided targets. In fact, targets located on the left hemifield gave rise to very similar patterns of response latencies when paired with both left and rightward face primes. Thus, the advantage for the right side of space seemed to arise only for combinations of rightward oriented primes paired with targets on the right hemifield as shown in direct comparisons. As expected, front-facing faces produced no differentiation in performance for target discrimination across hemifields, as these primes were absent of any directionality and therefore constituted our baseline condition. However, the similar performance following frontal faces across left and right targets does not necessarily imply symmetry. In fact, across all dependent measures (e.g., reaction times, first fixation onset) subtle differences can be observed between left and right hemifields. This means that front faces did not produce a mirrored performance towards left and right space but displayed a minor skewness. Biological determinants account for this result since the fusiform face area (FFA), defined by its selectivity for face perception, typically displays some lateralization^55^.

However, conclusions from Experiment 1, namely on the potential rightward bias in human attention scanning, should be draw with caution. Experiment 1 carries significant limitations by examining manual performance alone (i.e. response time) although traditional accounts on cueing tasks take behavioral performance as the signature response for attention allocation^39^. These conclusions are naturally bounded by the variables one can control in such a setup, which exclude participants oculomotor movements as a direct check for visual asymmetries.

These results are best interpreted in conjunction with supporting evidence provided by the false detections measure. A higher probability of obtaining false detections on the right (higher proportion of clicks in the right key) was found when participants were presented with right faces. In addition, the proportion distribution of false detections tells us that the response key was congruent with the directionality implied by the face prime. At the very least, this effect does suggest that facial primes affect attention orienting and shape consequent peripheral detection. Naïve to the actual manipulation in target-absent trials, participants unknowingly reported having seen the target in the hemifield corresponding to the orientation of the prior face cue. We believe that the right discrimination advantage obtained in response times, sustained by the congruency in false detections, hints at a systematic rightward asymmetry in the visual system.

In Experiment 2, we carefully monitored participants’ gaze movements and ensured that the face prime was attended to prior to target onset. Behavioral responses clearly demonstrate the robust congruency effect across both congruency conditions, an effect that was only obtained for the right congruent condition in experiment 1. A direct comparison between cue-target congruent pairs replicates the asymmetric right-side advantage obtained in Experiment 1. It is worth noting that the cueing effects produced by the frontal cues have overall resembled those observed for left-facing cues. Nevertheless, across measures of response time and first fixation onset in Experiment 2, for instance, it is possible to observe that front faces did not exactly mimic the performance obtained for leftward faces but have instead assumed intermediate values between left and right face perspectives. Although it is clear the pattern of effects follows that of leftward faces, front faces appear to have established an effect of their own, i.e. similar to but less pronounced than left faces. Arguably, the rationale behind the effect of frontal primes may come across as counterintuitive. It is reasonable to speculate that presenting baseline faces centrally has anchored the starting point of eye movement which one would assume to progress towards the right as in the case of reading, producing a carry-over effect and benefits on the right space. However, the systematic effect of front faces favoring the left space across the measures we report seems to suggest otherwise. We have hypothesized that since front faces are devoid of any inherent directionality and therefore should not prime lateralized attention. We propose that front faces, in lacking relevant directional content with the potential to trigger attention, are in fact comparable to an absence of prime. In this scenario, attention is likely to begin at center but to retrocede to the habitualized starting position of reading and writing routines (i.e. left) – although less markedly than in left faces.

Furthermore, a close inspection of participants’ gaze movements nicely revealed that initial saccadic behavior was congruent with the face prime. In a similar yet more reliable way than what false detections had already shown in Experiment 1, this report reiterates the capacity that face primes have to direct attention. We show that observed probabilities differ reliably from a uniform 50:50 distribution in left vs. right saccades, with right faces triggering rightward saccades to a greater probability than front- and left-facing faces. The first saccadic movement was also launched faster to the right region of space and therefore landed earlier in the right (versus left) located AOI following a rightward face.

Together, this set of studies provides evidence that attention is not equally distributed across hemifields. Biological influences of hemispheric specialization must surely exert some pressure on the lateralization of attention and should not be overlooked. Other genetic predispositions such as handedness, however, cannot account for our results. We did not observe differences in responding as a function of the left and rightly located targets on the keyboard. Thus, our data seems to suggest that attention is, to some extent, guided by prior expectations that are based on how language script unfolds, and movement progresses in space.

Although face cues were task-irrelevant, we show that a brief exposure to rightward faces suffices to counteract the expected spontaneous left-anchoring tendency for attention allocation. The proposal that faces both capture and hold attention and that gaze direction is best interpreted in conjunction with other cues such as head orientation is far from new^56–58^. Previous research has successfully shown that reading and writing routines largely contribute to the mapping of abstract concepts (i.e. time) which in turn biases orientation of spatial attention and primes congruent left/right responses^59^. Similarly, symmetry in conceptual congruency effects between abstract (i.e. past/future, good/bad, high/low status) and concrete (i.e. space, brightness, weight, temperature) domains can be modulated by attentional cueing^60^ and is linked to the exposure to asymmetric linguistic patterns and the degree to which these patterns are themselves asymmetric^61^.

Here, we take this rationale one step further by demonstrating that not only attention is spatially distributed following the left-to-right movement continuum but that there is an asymmetrical advantage for attending to the right side of space provided people are congruently cued. Although the leftward and rightward face manipulations used here refer to a generic abstract property – motion – attentional allocation did not produce bidirectional, similarly sized effects. In our view, our results, namely that visuomotor performance was not uniform but favored the right space, are due to the overlap with the habitualized rightward reading and writing routines of participants. This conclusion is supported by the fact that we have overruled handedness and stimulus–response compatibility as contributing factors. The observed right-sided discrimination facilitation is also beyond the stimulus–response compatibility effects found in other studies^13^ since the task at hand distinctively required target identification rather than mere location detection. Furthermore, we demonstrate that this tilted attention scanning goes well beyond the concrete activities of reading and writing and can be attained through means of very fundamental, language-absent, social cues like laterally averted faces. Indeed, rightward (versus leftward) faces have been reported to convey greater agency given that agency perceptions are largely drawn from the left-to-right movement continuum^51^. Arguably, perceptions inferred from our right profiles, although gender counterbalancing was controlled for, may have also added upon the left–right scanning practices of our participants, therefore partially accounting for the present results.

Evidently, the present research carries a number of limitations. First, to accurately draw conclusions on the underlying processes driving the attentional bias and asymmetric priming effects, we lack a sample of participants from right-to-left speaking countries whose attention is expected to flow on the opposite direction. Although reversal effects, albeit weaker, have been found for social representation of people^62,63^, aesthetic judgements^28^, and memory performance^64^, they have not been observed with the specific paradigm we tested here. Bidirectional readers could also provide important insight on the extent to which the attentional bias operates. In this case, the degree of the attentional bias is expected to be either negligible or a function of the more salient cultural background. While we believe that the systematic imbalance in responding behavior favoring rightward over leftward space (and therefore countering the biological proneness for the left anchoring of attention) suffices to argue that the convention for text direction exerts some form of attention control (as others have shown before us), only data from a sample habitualized with the reverse convention in this specific task would ensure indisputable evidence.

This work has important applied implications. These are particularly salient for the advertisement and marketing domains and for practitioners in fields relying on person perception, namely politics. These findings speak to the importance of tailoring the representation in space of any stimuli implying a sense of motion (i.e. cars, bicycles) according to the prevalent script on the receivers’ given culture. The placement of logos or other stimuli with non-dynamic properties also ought to consider the asymmetric distribution of attention in order to guarantee optimal capture of attention, processing fluency, memory, and recall^21,65^.

Altogether, these findings advance prior research by revealing that a fundamental cognitive process (i.e. attention), which was initially conceived as ‘culture free’ is susceptible to culturally maintained habits such as scanning practices derived from written text convention and has implications on how the environment is explored.

## Methods

### Experiment 1

#### Participants

Forty-five undergraduate students (37 females; *M*_*age*_ = 23.2, *SD* = 7.4; six self-reported left-handers) were recruited in exchange for course credit. Sample size was determined a priori using G*Power software package for within-subjects ANOVA with 80% power to detect an effect size of *η*_p_^2^ = 0.191 or similar as in earlier studies^66^. Participants were screened to normal or corrected-to-normal visual acuity. All participants were born in Portugal and the first language they had learned to read and write was a left-to-right language, that is, Portuguese. None of the participants had knowledge of or extended exposure to right-to-left languages. The experiment complied with the relevant guidelines of the institution and has thus received full ethics clearance from the Ethics Committee of ISPA – Instituto Universitário. An informed consent was collected from all participants.

#### Cues

Forty-two high-quality photos of unfamiliar faces previously piloted were used as cue stimuli. The face models signed an informed consent clearly stating that the photos in the different perspectives would feature in an online open access publication and would incorporate a photo dataset to be made available for research purposes. The photos were split into three sets of fourteen faces with fully averted profile to the left, the right, and front-facing perspective. Each set was counterbalanced for gender (7 male, 7 female). In order to avoid familiarity with the stimuli, the same face was never presented in two different face perspectives to a given participant. Six additional photos were used during the training phase following the same counterbalancing schema. The faces were presented with 10 × 10 cm and subtended 9.53° of visual angle. All face cues were centrally presented against a medium gray background.

#### Targets

The target stimuli consisted of two sets of five letters (4.77°) simultaneously presented to the left and the right side of the screen midpoint at ± 13.31° eccentricity, that is, in the near peripheral visual field. The target letter was either a *q* or a *p* embedded in one of the two letter sets (4 confounding letters and 1 target letter on one side of the screen; 5 confounding letters on the other) or no target letter (only distractors) was presented on both sides. Both confounding letters and target letters were kept constant across the experiment and their position within the target set was varied randomly across trials. This target setup, that is one of two possible target letters appearing on either side, required discrimination rather than mere detection^67^. This prevented participants to assume target location if the target was not present in the letter set they first attended to. Thus, if participants gaze towards the right and the target is not present, then it necessarily means that the target letter is on the left. However, this information is not sufficient to infer which of the two target letters (*p* or *q*) is the correct one.

Moreover, by introducing a target-absent condition, we were able to explore the extent to which false detections are a function of distinct head orientations.

#### Apparatus

The task was programmed using E-prime 2.0 (copyright 2010, Psychology Software Tools, Inc.). The stimuli were displayed on an Asus VX238H 23″ Full HD LED monitor (1920 × 1080) and the task was run on a Dell OptiPlex 755 with a refresh rate of 60 Hz. The monitor was placed at the viewing distance of 60 cm. A Cedrus RB-540 response pad recorded participants response times and error rates. Participants responded to the target by pressing the left key (*q*) or right key (*p*).

#### Design

The design was a 3 (face orientation: left vs. front vs. right) × 3 (target letter position: left vs. right vs. no target) × 3 (response interval: 700 ms vs. 1000 ms vs. 1300 ms) within-subjects’ factors. All measures, manipulations, and exclusions in this study are disclosed.

Valid (congruent), invalid (incongruent) and target-absent trials were kept constant across the experiment so that face cues were non-informative of target location. All factor combinations were equiprobable and presented equally often throughout the experiment. The front-facing faces constituted the baseline and had the exact same target distribution. Target type *q* and *p* was counterbalanced within face orientation and validity.

#### Procedure

The task was administrated in multiple sessions in the research laboratory of the University. Participants were instructed to attend to the target to generate a response because faces were uninformative of the target location. The general instruction was a speed-accuracy one. Participants placed their index fingers on the respective response keys and were asked to press them as soon as they located the target letter *p* or *q*. Importantly, participants were not told that in some trials there would be no target.

**Figure 6:**
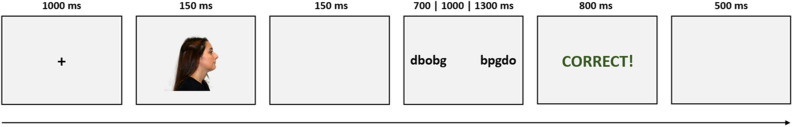
Procedure schema with an example of a cue-target rightward congruent trial.

The task consisted of a variation of an attention orienting paradigm. The trial sequence commenced with a fixation cross (0.3° × 0.3°) at the center of the display for 1000 ms followed by the presentation of the face cue for 150 ms. A blank screen for stimulus onset asynchrony followed the cue and lasted 150 ms. The two letter strings were presented on the left and right sides of the display prompting an answer by the participant. Participants were requested to answer within a brief response window (see below). If participants failed to discriminate the target letter within the given response window, then it constituted a missing trial. A screen with a feedback message followed for 800 ms informing participants about the accuracy of their response or instructing them to be faster in case that the response time had elapsed. A blank screen was then presented for 500 ms and a new trial began (Fig. 6).

The response window durations varied between 700 ms, 1000 ms, and 1300 ms. The reason for this manipulation was two-fold. For one, preliminary pilots revealed that the task was highly demanding upon selective attention and carried a high perceptual load due to the large the number of items to be processed. Second, these intervals were chosen to allow us to explore within participants how reaction time in discrimination evolves over time. By relying on three response length intervals, we could pinpoint whether discrimination occurs at the same point in time or not, despite the interval given to respond.

Each block consisted of 42 trials. The experiment comprised 6 blocks resulting in a total of 252 trials, 84 per response window. The three response intervals were randomized across trials. Prior to the main experiment, participants completed 12 practice trials. The average completion time of the experiment was 20 min.

### Experiment 2

#### Participants

Forty participants (36 female, *M*_Age_ = 25.35, *SD* = 8.13; nine self-reported left-handed) took part in the experiment in return for course credit or a commercial voucher. All participants were screened to normal or corrected-to-normal visual acuity. All participants were native Portuguese speakers. Informed consent was collected from all participants and the experiment was performed in accordance with the relevant guidelines and approved by the Ethics Committee of ISPA – Instituto Universitário.

#### Apparatus

The task was programmed using Experiment Builder (Version 1.10.1630, SR Research, 2016). The stimuli were displayed on an Asus VX238H 23″ Full HD LED monitor (1920 × 1080) driven by a Dell OptiPlex 755 with a refresh rate of 60 Hz. An Eyelink 1000 plus eye tracker (SR Research) with a sampling rate of a 1000 Hz was calibrated to the participants’ dominant eye, but viewing was binocular. Calibration was performed with the standard nine-point calibration procedure, resulting in a reported interval of 0.25°—0.5° average accuracy for all points. Calibration was repeated if the error at any point was higher than 1°. A chin and forehead rest were used to restrict participants’ head movements and to control for the viewing distance to the screen at 60 cm. Responses were collected using the keys *q* and *p* on a standard keyboard.

#### Procedure

The task was administrated in single sessions in a dimly lit room in the research laboratory of the University. The general procedure, block composition and counterbalancing were the same as in Experiment 1. However, the fixation cross marking the beginning of each trial was replaced by a drift check, which only triggered the next trial if participants focused on it for a minimum of 1000 ms. Each drift check prior to trial onset was manually accepted by the experimenter. This procedure ensured that the starting point of eye movement for each trial was in the center of the display, thus preventing attention to be oriented elsewhere prior to cue onset. Between each of the six blocks of 42 trials, participants took a self-paced break followed by a recalibration. The experiment lasted approximately 40 min.

## Data availability

The raw and processed datasets generated and analyzed during the current study as well as the stimuli set are available in the Open Science Framework repository, https://osf.io/j7y4x/.
